# Development of propolis, hyaluronic acid, and vitamin K nano-emulsion for the treatment of second-degree burns in albino rats

**DOI:** 10.1186/s12906-024-04377-6

**Published:** 2024-02-16

**Authors:** Marwan Elsamman, Ola M. El-borady, Mohanad M. Nasr, Zeinab Al-Amgad, Asmaa A. Metwally

**Affiliations:** 1 Faculty of Biotechnology, October University for Modern Science and Arts (MSA), 6th October, Giza, Egypt; 2 Institute of Nanoscience and Nanotechnology, Kafr Elsheikh University, Kafr Elsheikh, 33516 Egypt; 3 General Authority for Veterinary Services, Qena Veterinary Directorate, Qena, 83523 Egypt; 4 Department of Surgery, Anesthesiology, and Radiology, Faculty of Veterinary Medicine, Aswan University, Aswan, 81528 Egypt

**Keywords:** Burns, Propolis, Hyaluronic acid, Vitamin K, Nano-emulsion, Wound healing assay

## Abstract

Burns are the fourth most common type of injury worldwide. Many patients also suffer numerous infections and complications that impair the burn healing process, which makes the treatment of burns a challenge. This study aimed to prepare and characterize nano-emulsion (NE) of propolis, hyaluronic acid, and vitamin K for treatment of second-degree burns. High-Pressure Liquid Chromatography (HPLC) was used for the qualitative assessment of the phenolic and flavonoid contents in crude propolis. The structural, optical, and morphological characterization, besides the antimicrobial, antioxidant, cytotoxicity, *in-vitro*, and *in-vivo* wound healing activities were evaluated. For *in-vivo* study, 30 adult male albino rats were divided randomly into control and treated groups, which were treated with normal saline (0.9%), and NE, respectively. The wounds were examined clinicopathologically on the 3rd, 7th, and 14th days. The NE revealed the formation of a mesh-like structure with a size range of 80–180 nm and a 21.6 ± 6.22 mV zeta potential. The IC_50_ of NE was 22.29 μg/ml. Also, the NE showed antioxidant and antimicrobial activity against *Escherichia coli, Pseudomonas aeruginosa* and *Staphylococcus aureus*. The *in-vitro* investigation of the NE on normal human skin fibroblasts using scratch assay proved an acceleration for wound healing. The treated rats showed improved wound healing clinically and pathologically and wound contraction percent (WC %) was 98.13% at 14th day, also increased epithelization, fibrous tissue formation, collagen deposition, and angiogenesis compared to the control. It could be concluded that the prepared NE possesses antimicrobial, antioxidant, and healing effect in the treatment of second-degree burns.

## Introduction

The skin serves as a vital and effective barrier to the surroundings. It protects the body from germs, xenobiotics, and dehydration, among other things [1, 2]. Moreover, it defends against a range of hazards, including thermal, chemical, and UV radiation besides controlling body temperature, boosting metabolic activities, and synthesizing vitamin D, as well as keeping the body in contact with the surroundings through a variety of nerve endings [3]. However, this essential barrier can be severely injured by burns which are the most severe kind of soft tissue injuries [4].

The care of burn patients was extremely limited until the first half of the twentieth century, and patients frequently perished as a result of hypervolemic shock in the early days following injury. Regenerative medicine, burn treatment, and pharmacology all saw rapid advancements in the second half of the twentieth century. Nonetheless, treating burn injuries is still a challenge [5]. Burn wounds are classified as superficial (first degree), partial-thickness (second degree), or full-thickness (third degree) based on their depth, which dictates the therapy required for them to recover successfully [6].

Friction, cold, heat, radiation, chemical or electric causes can all lead to burn injuries and cause diverse physiological and pathophysiological reactions. All burn injuries result in tissue damage and cause coagulative necrosis [7]. The cause and depth of damage are used to classify thermal injuries. Fire, scalding, and contact with hot or cold objects are all causative agents. They promote tissue damage through energy transfer [8].

Wound healing is essential for survival, as it restores the skin’s integrity and protects the individual from infection and dehydration. The process of wound healing is a well-coordinated chain of events that leads to the restoration of wounded tissues and the creation of scars [9]. Burn-specific therapy regimens must be created, as burn wound healing is more complicated than other skin injuries [6].

Infection is a major concern for burn victims as it triggers a strong immune response, which can lead to sepsis or septic shock, which in turn slows wound healing. Furthermore, sepsis and multi-organ failure are the primary causes of mortality after a severe burn [10]. Exogenous bacteria and fungi, as well as typical microorganisms of the regular skin flora, can quickly obtain access to underlying tissues, which provide a humid, warm, and nutrient-rich environment for their development. When healing is delayed, the wound’s typical microbiota altars, and more aggressive bacteria strains take up residence. As a result, an open wound might be an ideal environment for microbial growth and colonization [11].

Different material including artificial skin [12], polymers [13], hydrogels [14], and hybrid materials [15, 16] are explored for acceleration of burn healing process [17]. Also, gelatin and alginate based wound dressings show strong potential. Both polymers are modified by introducing photocrosslinkable functionalities and combined to hydrogel films (gel-MA/alg-MA) [18]***.***

Currently, silver sulfadiazine cream Is used as a topical therapy for burn wound infection. This medication has clinical gaps and may be ineffective against multidrug-resistant pathogens present in burn wounds in humans [19].

The use of nanoparticles as a drug delivery mechanism has been recognized and researched. Chitosan and silver NPs composed film with ultralow silver level and without aggregation of AgNPs was used for dressing of thermal burn wounds [17].

Nano-emulsion therapy is a potential therapeutic option for burn wounds. The development of a topical antibacterial therapy that also controls the skin’s inflammatory response to burn injury and prevents partial-thickness burns from converting to full-thickness burns might dramatically enhance early treatment for burn patients [20].

Biogenic medicines having antimicrobial, regenerative, and analgesic qualities are becoming more important in the treatment of burn wounds. Apitherapeutics, such as propolis and honey products gathered and processed by honey bees, possess these qualities [21]. Besides their antimicrobial activity, the application of propolis to wounds stimulates wound bed matrix remodeling, leading to faster repair of the burned tissue, which could be observed in the changes in the extracellular matrix. This could be due to its ability to reduce lipid peroxidation and prevent necrosis [22].

Propolis possesses anti-bacterial, anti-inflammatory, antioxidant, anti-cancer, and immunomodulatory properties. It has been utilized in a range of products, including wound healing ointments and creams, as well as the treatment of burns, skin, and ulcers [23–25].

Hyaluronic acid (HA) is a component of the skin’s extracellular matrix and other connective tissues. Because of its hygroscopic power, which is connected to its high molecular weight and negative charge, it has a substantial water-binding capacity. It contributes to the preservation of a moist environment conducive to the re-epithelialization process [26]. Several *in-vitro* and *in-vivo* experiments on animals and people have proven that applying hyaluronic acid topically to wounds enhances healing and reduces healing time [27]. As part of cell proliferation and migration, HA plays two critical roles in wound healing. First, HA acts as a temporary foundation in the early phases of a wound. This structure aids in the dispersion of nutritional supplies and the removal of waste products from cell metabolism from the wound. Second, and most critically, HA plays a crucial role in keratinocyte proliferation and migration [28].

Vitamin K is a fat-soluble vitamin that is essential for living organisms’ coagulation mechanisms [29, 30]. Vitamin K is required for the carboxylation of certain glutamic acid residues to generate carboxyglutamic acid, and thus plays a role in growth regulation, signal transduction, proliferation, apoptosis, and phagocytosis [31]. A study led by [32] examining the wound healing properties of topical vitamin K on full thickness wounds in rats, reported promising results. When compared to the control group, the effects of topical vitamin K demonstrated substantial healing in wound contraction, epithelialization time, hydroxyproline content, and tensile strength. Vitamin K also showed effectiveness in histopathological examinations.

The main aim of the current study was to combine for the first time the three materials (propolis, hyaluronic acid, and vitamin K) in the form of a nano-emulsion, then evaluate the wound healing potential of the new NE through *in-vitro* and *in-vivo* studies, antioxidant, and antimicrobial activity against commonly isolated bacteria from burns.

## Materials and methods

### Propolis sample, solvent and chemical

Crude propolis was collected from the farm of the Faculty of Agriculture, South Valley University, Qena, Egypt; Vitamin K, was purchased from AMRIYA Pharmaceuticals; hyaluronic acid, Ethanol, and Tween 80 were purchased from Sigma-Aldrich; human skin fibroblast cell lines as well as *Staphylococcus aureus* ATCC 25923, *Escherichia coli* ATCC 8739, and *Pseudomonas aeruginosa* ATCC 27853 were all obtained from Nawah Scientific Inc., (Mokatam, Cairo, Egypt).

### Qualitative Phenolic & Flavonoid Assessment

The qualitative identification of phenolic and flavonoid compounds in Propolis was carried out by HPLC by Nawah Scientific Inc. (Mokatam, Cairo, Egypt). A Waters 2690 Alliance HPLC system equipped with Waters 996 photodiode array detector, Milford, CT, USA. Laminar Flow (model 1386, Thermo Fisher Scientific, Waltham, MA, USA). A stock solution of 10 different standards in methanol was prepared and filtered using a 0.22 μm syringe filter, then 10 μl was injected. 137.8 mg/ml of propolis were accurately weighted and sonicated for 15 min, filtered using a 0.22 μm Nylon syringe filter, then 10 μl was injected. The gradient method that was eventually chosen following a series of preliminary studies uses a mixture of 0.1% Phosphoric acid in water: Acetonitrile (mobile phase). The total runtime of the method was 80 minutes. The absorbance was measured at 280 nm. The total phenolic content (mg/mL) was calculated using the standards; Gallic acid, chlorogenic acid, ellagic acid, and caffeic acid. The total flavonoid content (mg/mL) was calculated using the following standards; catechin, rutin, hesperidin, apigenin, kaempferol, and quercetin.

### Preparation of Nano-emulsion [33]

The prepared NE consists of propolis, hyaluronic acid, and vitamin K, and was synthesized following the emulsification-solvent technique (O/W) using a probe ultrasonicator. Initially, the aqueous phase was prepared by dissolving 0.2 g of hyaluronic acid in 40 ml distilled water using a magnetic stirrer for 10 minutes. In parallel, the oil phase was prepared in another dry beaker by dissolving 0.5 g propolis in 10 ml absolute ethanol and stirring for 1 hour at room temperature followed by filtration using Whatman filter paper No 1. 20 mg vitamin K and 2 ml tween 80 (surfactant) were added to the filtered propolis. Finally, the oil phase solution was added dropwise to the aqueous phase solution and emulsified using a Vibra cell™ ultrasonicator (Newton, Massachusetts, USA), which was set to 20,000 Hz for 20 minutes.

### Characterization of the Nano-emulsion

The nano-emulsion’s size and morphology were investigated using high-resolution electron microscopy (HR-TEM), where the images were taken with the transmission electron microscope (JOEL JEM-2010) operated at an acceleration voltage of 200 kV connected to a Gatan digital camera model Erlangshen ES500.

The zeta potential of the prepared sample was measured by a Malvern Zetasizer model Nano ZS-90 operating at 25 °C. The Fourier-Transform Infrared (FTIR) spectra were used for demonstration of the structural composition of the NE and was detected using a JASCO spectrometer in a scanning range of 4000–400 cm^−1^ using KBr as a reference.

### DPPH assay

The free radical scavenging activity was examined via the DPPH (2,2-diphenyl-1-picryl-hydrazyl-hydrate) assay, where 1 ml of the emulsion was mixed with 1000 μl of DPPH (0.2 mM) along with control DPPH, which doesn’t contain any nanoparticles. These mixtures were blended for 3 min in dark conditions at ambient temperature. Then, after 20 min, the radical concentration was determined by measuring the decrease in absorbance percentage of the mixture at 517 nm wavelength. The control was set up as over, and the sample was utilized for the gauge revision. The change in the absorbance of the sample was estimated at 517 nm. Vitamin C (ascorbic acid) was used as a reference. The free radical scavenging activity was calculated using the following equation:

$$\textit{Radical scavenging activity}=((\textit{Control absorbance- Sample absorbance})/\textit{Control absorbance})\times100$$

### Antimicrobial assays

The antimicrobial activity of the prepared NE was tested against ***Staphylococcus aureus*** *(ATCC 25923),* ***Escherichia coli*** *(ATCC 8739), and* ***Pseudomonas aeruginosa*** *(ATCC 27853)*, which are the most commonly isolated bacteria from burns [34]. The inoculums were prepared via the Colony suspension method and tested using Broth Macrodilution method.

#### Colony suspension method

A disc of each *Staphylococcus aureus (ATCC 25923), Escherichia coli (ATCC 8739), and Pseudomonas aeruginosa (ATCC 27853)* were achieved according to Fahmy et al. [35] were inoculated into 100 ml of tryptic soy broth medium and incubated at 37 °C ± 1.0 for 24 h. For preparation of fresh (18–24 h) culture agar plate, a loopful from the broth was streaked onto Tryptic Soy Agar medium, then incubated at the same previous temperature. A direct sterile saline solution was prepared by inoculating Three or four colonies (from the organism plate), and the suspension was adjusted to achieve a turbidity equivalent to a 0.5 McFarland standard of each strain using DensiCHEK© optical device (BioMérieux, Lyons, France). The adjustment results in a suspension containing approximately 1–2 X 10^8^ CFU/mL. The suspension was diluted by inoculating 1.0 ml of inoculum into 150 ml of Muller Hinton broth, which resulted in approximately a concentration of 1.0 X 10^6^ CFU/ml. Any subsequent 1:2 dilution shall result in 5.0 X 10^5^ CFU/well.

#### Broth macrodilution method

In 24 wells plate, 1.0 ml Muller Hinton broth (MHB) was inoculated into all testing wells (Escaping the first well). Then, 2.0 ml from the sample was directly inoculated in the first well (without dilution). Next, 1.0 ml was aspirated and transferred to the next well and mixed well to make 1:2 dilution, then 1.0 ml was aspirated from the 1:2 dilution using the new tip and mixed with 1.0 ml broth (1:4 dilution). Eight dilutions for each strain were prepared and inoculated in a 24-well plate. Followed by 1.0 ml of the prepared inoculum being added to each well, resulted in a final concentration of 5.0 X 10^5^ CFU/ml. Another 1.0 ml from each bacterial suspension was diluted and cultured to confirm inoculum density. All plates were incubated at 37 °C ± 1.0 °C for 24.0 ± 2.0 h for determination of MBC. After the incubation period, a loopful of each clear well was streaked onto the Tryptic soy agar plate. The lowest concentration with the absence of growth was considered as MBC/MFC.

### Cytotoxicity assessment using sulforhodamine B (SRB) assay

A human skin fibroblast cell line (HSF) obtained from Nawah Scientific Inc., (Mokatam, Cairo, Egypt) was used for the cytotoxicity assay. HSF was maintained in Dulbecco’s Modified Eagle Medium (DMEM) media supplemented with 100 mg/mL of streptomycin, 100 units/mL of penicillin, and 10% heat-inactivated fetal bovine serum in a humidified 5% (v/v) CO_2_ atmosphere at 37 °C [36, 37]**.** The cell viability was assessed by SRB assay. Aliquots of 100 μL cell suspension (5 × 10^3^ cells) were placed in 96-well plates and incubated in media for 24 hr. Cells were treated with another aliquot of 100 μL media containing the prepared NE at various concentrations. After 72 h of drug exposure, cells were fixed by replacing the media with 150 μL of 10% Trichloroacetic acid (TCA) and incubated at 4 °C for 1 h. The TCA solution was removed, and the cells were washed five times with distilled water. Aliquots of 70 μL SRB solution (0.4% w/v) were added and incubated in a dark place at room temperature for 10 min. Plates were washed three times with 1% acetic acid and allowed to air-dry overnight. Then, 150 μL of TRIS (10 mM) was added to dissolve the protein-bound SRB stain; the absorbance was measured at 540 nm using a BMG LABTECH®- FLUOstar Omega microplate reader (Ortenberg, Germany).

### Wound scratch assay

The human skin fibroblast cell lines were prepared as mentioned before. For the wound scratch assay, the cells were plated at a density of 2 × 10^5^/well onto a coated 12-well plate and cultured overnight in 5% FBS-DMEM at 37 °C and 5% CO_**2**_. On the next day, horizontal scratches were introduced into the confluent monolayer; the plate was thoroughly washed with PBS, and control wells were replenished with fresh medium, while test wells were treated with fresh media containing the NE. Images were taken using an inverted microscope at the indicated time intervals. The plate was incubated at 37 °C with 5% CO_**2**_ in between time points. The acquired images were displayed below and analyzed by MII Image View software version 3.7. The wound closure percentage was calculated using the following equation:

$$\textbf{Wound}\ \textbf{closure}\;\%:\left(\textbf{At}=\textbf{0}\ \textbf{h}\textbf{r}-\textbf{At}=\boldsymbol{\Delta} \textbf{h}/\textbf{At}=\textbf{0}\ \textbf{h}\right)\times \textbf{100}$$

Where At = 0 hr is the average area of the wound measured immediately after scratching (time zero), and At = Δh is the average area of the wound measured h hours after the scratch is performed.

### *In-vivo* study

#### Animals

Thirty adult male rats weighted 180–230 g body weight and 2–3 months old were purchased from the National Research Center’s (NRC) animal housing unit. The animals were housed in groups in plastic cages for a week before the experiment for acclimatization and adaptation under standard laboratory conditions of controlled room temperature (21–25 °C), humidity (42–55%), and a 12-hour light-dark cycle. They were given balanced ration with water freely accessed. The rats were kept for 3 weeks for adaptation before the induction of the experiment.

#### Burns creation

The rats were anesthetized intraperitoneally (IP) using Ketamine (70 mg/kg)/ Xylazine (7 mg/kg) combination. The second-degree burns were created on the back using a circular 3*3 cm brass plate that was heated to 100 °C and pressed to the skin for 5 seconds [38]. The rats were divided randomly into control and treated groups (*n* = 15 rats for each), the burns of the control group were treated with normal saline (0.9%). While the burns of the treated group were treated with NE. On the 3rd, 7th, and 14th days postoperatively, burns from different groups were inspected, photographed, and the burn diameter was measured with a digital caliper for visual comparison before and after clearance of the burn area. Also, WC % was calculated as follows:

$$\textbf{Wound}\ \textbf{Surface}\ \textbf{Area}\ \left(\textbf{WSA}\right)=\uppi {\textrm{r}}^2\ \left({\textrm{cm}}^2\right)$$

$$\textbf{Wound}\ \textbf{Surface}\ \textbf{Area}\ \textbf{percent}\ \left(\textbf{WSA}\%\right)=\frac{{\textrm{WSA}}_{\textrm{Test}}}{{\textrm{WSA}}_{\textrm{Control}}}\times 100$$

$$\left(\textbf{WC}\%\right)=100\%-\textrm{WSA}\%$$

Where π (Pi) is a mathematical constant (22/7) and r^2^ is the radius squared as the burn area was 3*3 cm.

#### Histopathological study

Burns samples were dissected from both groups at the 3rd, 7th, and 14th days post-treatment after euthanasia of rats with an overdose of Ketamine/ Xylazine combination [39], then fixed in 10% buffered formalin. The fixed samples were processed for paraffin embedding staining. Sections at 5 μm thickness were cut and stained with Haematoxylin and Eosin (HE) for morphological examination and stained with Crossman trichrome for collagen fibers detection. Additionally, fibrous tissue formation, cell mitosis, tissue granulation, re-epithelization, angiogenesis, fibroblast, neovascularization, ulcerative necrosis with cell debris, inflammatory edema, and inflammatory cells infiltration scores were detected.

### Statistical analysis

The Statistical Package for Social Sciences (SPSS, ver. 20) was used for data analysis. The data were analyzed using one-way ANOVA. All the values were expressed as mean ± Standard Deviation (SD) and considered significant when *p* ≤ 0.05. All the graphs were expressed in Excel.

## Results & discussion

Burns from different mishaps are common among Egyptians, yet they may go unreported or undocumented. The lack of competent burn facilities in Egypt, particularly in Upper Egypt, continues to be a major issue that claims the lives of many burn patients [40]. Therefore, the present study aimed to evaluate the antimicrobial, antioxidant, and *in-vitro* wound healing as well as *in-vivo* burn healing effect of the prepared nano-emulsion of propolis, hyaluronic acid, and vitamin K.

### Qualitative Phenolic & Flavonoid Assessment of Propolis using HPLC

**Fig. 1:**
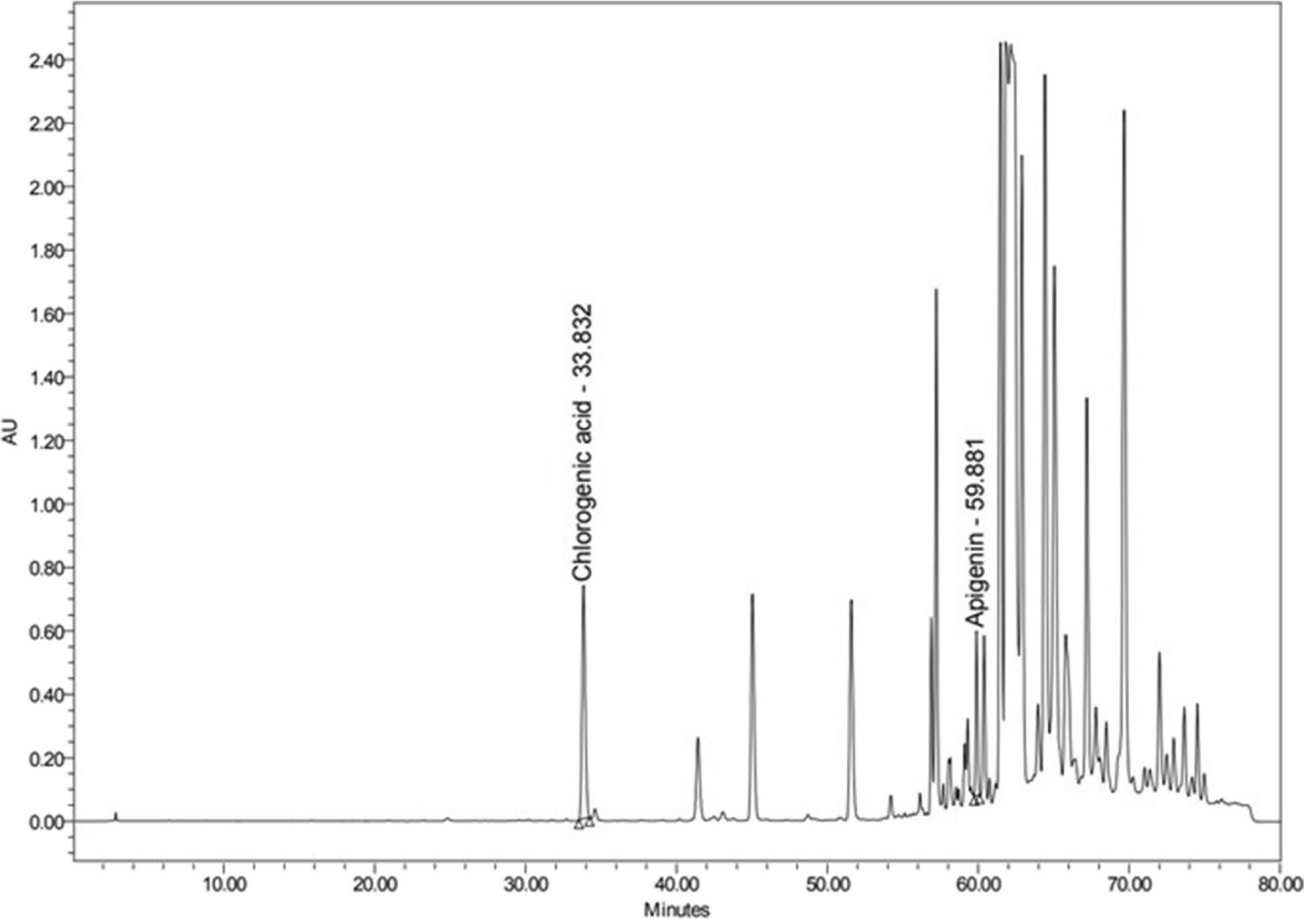
Qualitative phenolics & flavonoids assessment of crude propolis using HPLC Apigenin, detected at retention times 33.832 and 59.881, respectively

In the present study, HPLC was used to analyze the phenolics and flavonoids present in crude propolis, which was found to contain Chlorogenic acid and Apigenin at retention times 33.832 and 59.881, respectively, and both having wound healing capabilities (Fig. 1**)**. Most of the antioxidant action in plants or plant-based products is attributed to phenolic compounds, the largest category of phytochemicals. They make up the biggest class of naturally occurring phenolic compounds and may be found in both their free form and as glycosides [41]. According to Chen et al. [42], the capacity of Chlorogenic acid to promote collagen production through the up-regulation of critical players such as tumor necrosis factor-α and transforming growth factor-β1 in different stages of wound healing process can speed the process of excision wound healing. Moreover, a study led by Rajab et al. [43] on the role of Apigenin-based cream on wound healing in a rabbit model, they reported that the Apigenin group demonstrated considerably improved wound healing ability in rabbit skin; wound size and wound contraction ratio were better in the Apigenin group compared to the controls.

Interestingly, a study carried out by Kapare et al. [44]**.** on Indian propolis revealed that propolis also comprised on Apigenin. However, they also found that it contained caffeic acid, quercetin and caffeic acid phenethyl ester, which were not detected in the current study. Nevertheless, this can be attributed to the difference in the source of the propolis samples. According to Wahyuni and Riendriasari [45], the diversity of flavonoid content of propolis differs depending on bee species, duration of extraction, and propolis hive part.

### Characterization of nano-emulsion

#### Transmission Electron microscope (TEM)

**Fig. 2:**
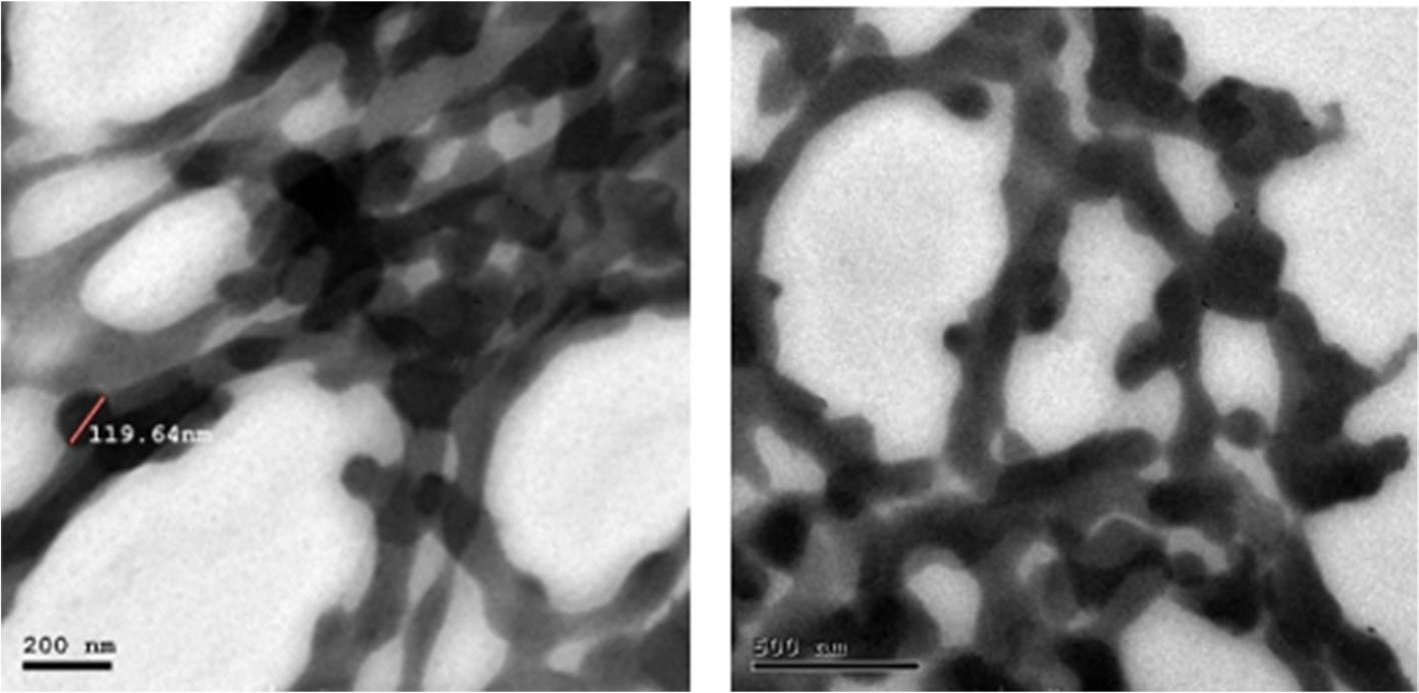
TEM images for the synthesized propolis nano-emulsion (the two images at different magnification scales and different spots)

**Fig. 3:**
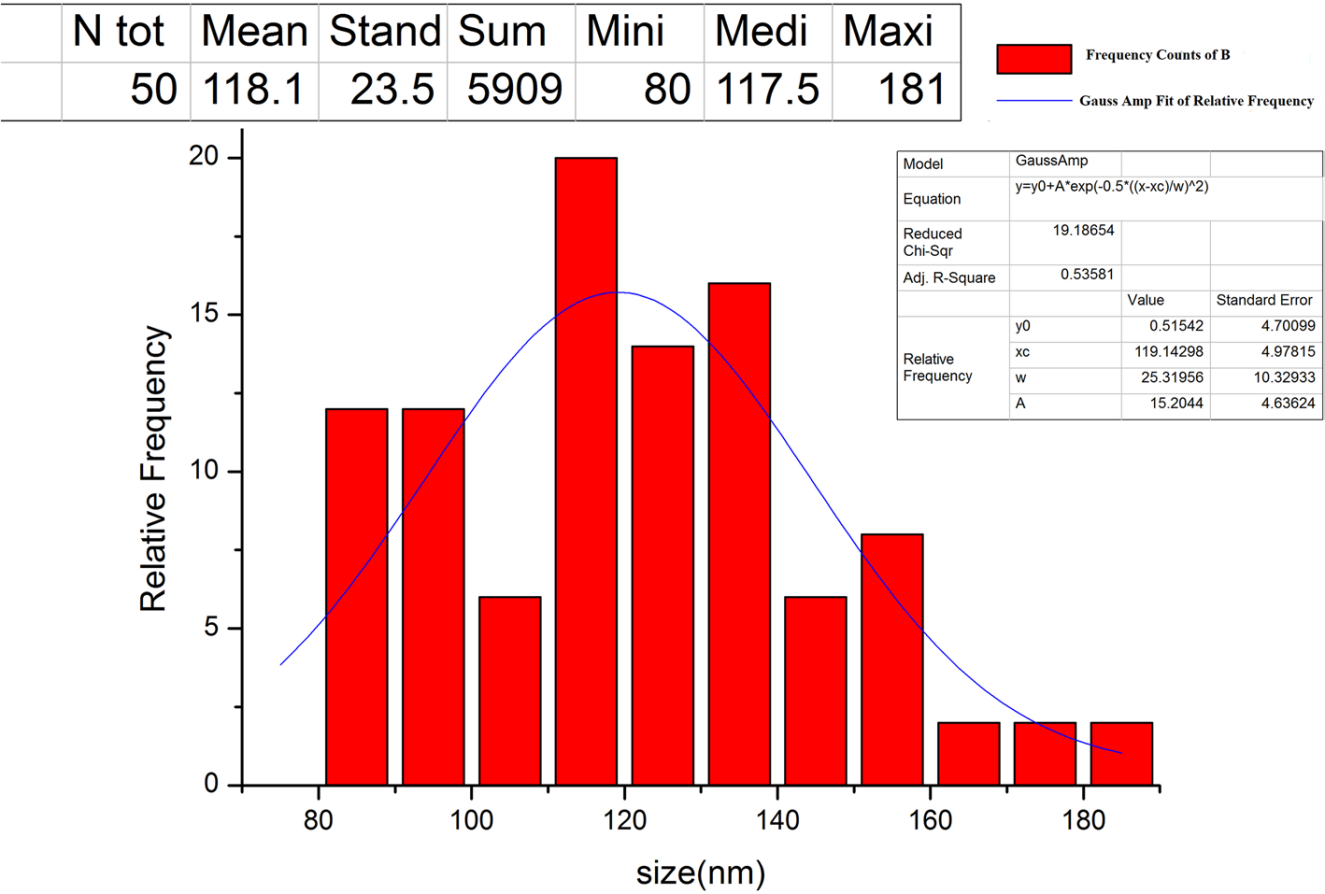
The histogram represents the particle sizes of the propolis nano-emulsion

The TEM micrograph of the prepared NE showed that the NE droplet has somewhat of a mesh form **(**Fig. 2**).** The size range of NE was found to be between 80 nm and 180 nm, with the mean being 118.1 nm, as indicated by the TEM histogram in Fig. 3**.**

#### Fourier-transform infrared (FTIR) spectroscopy

**Fig. 4:**
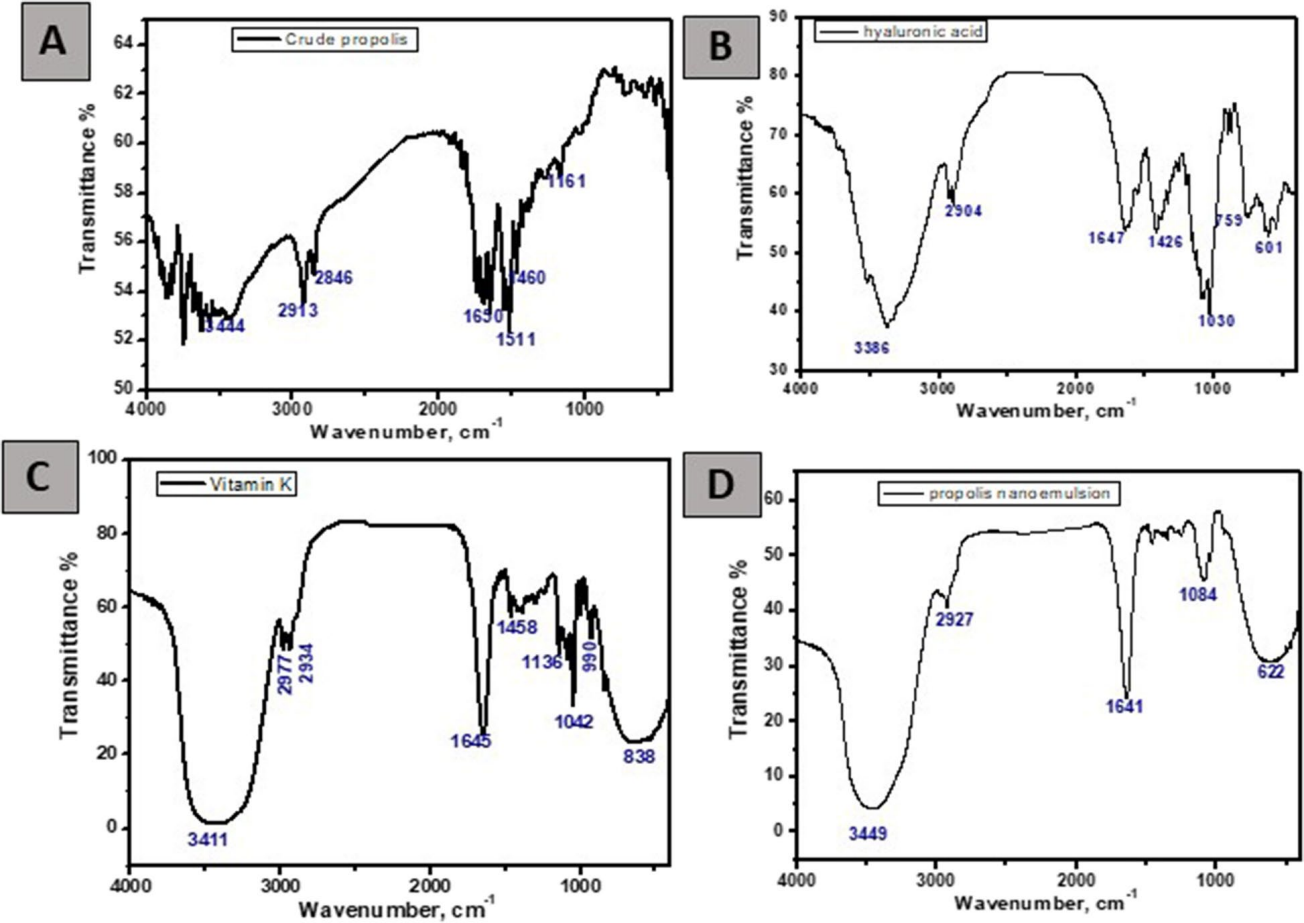
FT-IR spectra of: **A** crude propolis, **B** hyaluronic acid, **C** vitamin K, **D** propolis nano-emulsion

The FTIR spectra of the crude propolis, hyaluronic acid, vitamin K, and NE were displayed in Fig. 4. A great similarity was observed between them, mainly when the peaks at 3449 cm^−1^, 2927 cm^−1^, 1641 cm^−1^, 1084 cm^−1^, and 622 cm^−1^ were analyzed.

The presence of both organic and inorganic chemicals in a sample was determined by FTIR. The specific chemical groups that predominate in a sample were identified using spectrum data in the automated program of spectroscopy depending on the infrared absorption frequency range of 600–4000 cm^−1^ [46].

In the current study, the propolis nano-emulsion showed a peak at 3449 cm^−1^ assigned to N-H stretching of an aliphatic primary amine, a peak at 2927 cm^−1^ assigned to C-H stretching of an alkane, another peak at 1641 cm^−1^ assigned to C=C stretching of an alkene, a peak at 1084 cm^−1^ assigned to C-O stretching which may be attributed to some ethanol residue, as ethanol was used in the preparation of the emulsion. This agreed with a study conducted by Toledo et al. [47]**,** in which they prepared propolis biofilms with a 50:50 ratio of propolis and ethanol, upon the analysis of the propolis films using FTIR, they also found a peak at 1084 cm^−1^ which attributed to ethanol residue. Finally, a peak was found at 622 cm^−1^ assigned to the C-Br stretching of a halo compound.

#### Zeta potential & size distribution

**Fig. 5:**
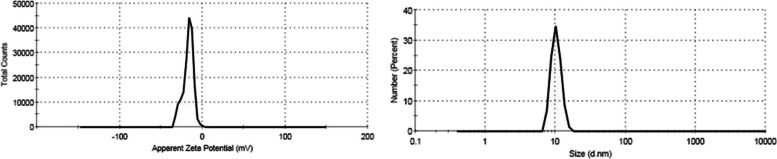
**A** Zeta Potential of Propolis Nano-emulsion, **B** Size distribution by number of the propolis nano-emulsion

Zeta potential provides information about the surface charging behavior in contact with water-based electrolytes [48]. In the current study, the prepared NE had a negative zeta potential of − 21.6 ± 6.22 mV (Fig. 5**)**, which was strong enough to prevent nanoparticles aggregation. This number denotes a stable and distributed dispersion of nanoparticles with no propensity to agglomerate in a short period of time. As a result, the nano-emulsion’s stability can be related to a negative surface charge, which causes particles to repel one another [49] and to the steric effect stabilization presented by large molecules in the NE. This agreed with the results of a study conducted by Kazemi et al. [50] who assessed the interaction of free, glycated, and fructated hemoglobin with propolis nanoparticles that provided zeta potential of propolis NPs of − 20.57 ± 1.4 mV. Our result was also compared with a study conducted by Hegazi et al. [51] on the antibacterial efficacy of Egyptian propolis-encapsulated alginate NPs against several harmful bacterial strains; the measured zeta potential of the propolis NPs was − 28.10 ± 5.54 mV.

Furthermore, the Z-average size of 10.35 ± 1.719 d. nm (Fig. 5) and the polydispersity index (PDI) value of 0.226 indicated the monodispersity of the particles of NE. The viscosity of NE was 0.8872 cP which attributed to the presence of hyaluronic acid [52].

#### The UV-Vis absorption spectroscopy

**Fig. 6:**
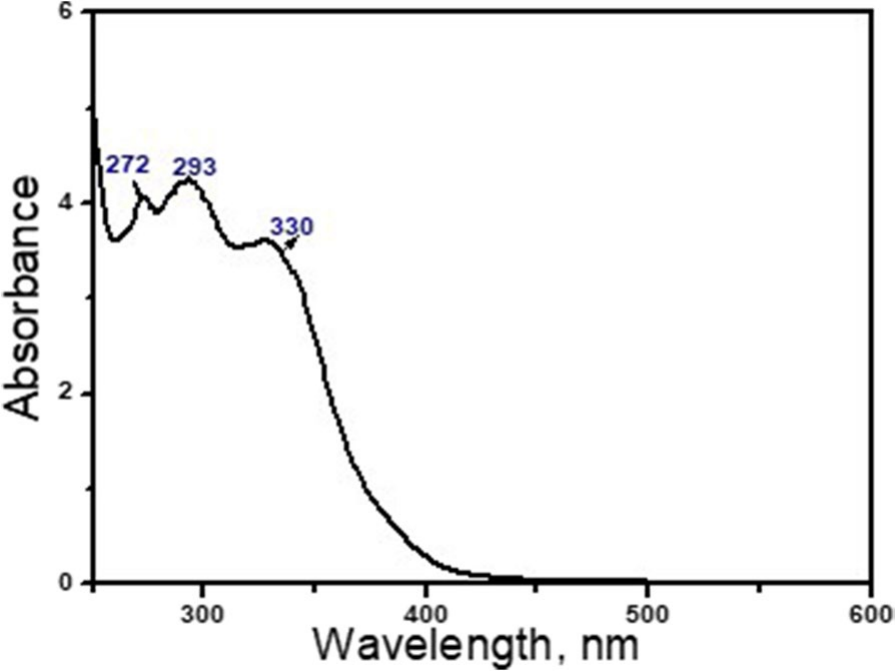
The absorption spectrum of the synthesized propolis nano-emulsion

The absorption spectrum of the synthesized propolis NE is shown in Fig. 6. The spectrum revealed three short peaks centered at 272 nm, 393 nm, and 330 nm, which could be attributed to π-conjugated aliphatic and aromatic compounds as well as aromatic acids in the propolis (the main component in the NE), as previously investigated by Abdullah et al. [53] Furthermore, the active ingredients in various bee propolis documented in the literature [54] are primarily the flavonoid and phenolic compounds, such as quercetin, caffeic acid, coumaric acid, tocopherol, benzoic acid, ferulic acid, cinnamic acid, vanilic acid, and others. Additionally, the hyaluronic acid peaks may appear in the same region of propolis but may be merged under the propolis spectrum region. While, as described by Pan et al. [55], the hyaluronic acid showed an absorption peak at below 300 nm.

### Cytotoxicity assessment using SRB assay

**Fig. 7:**
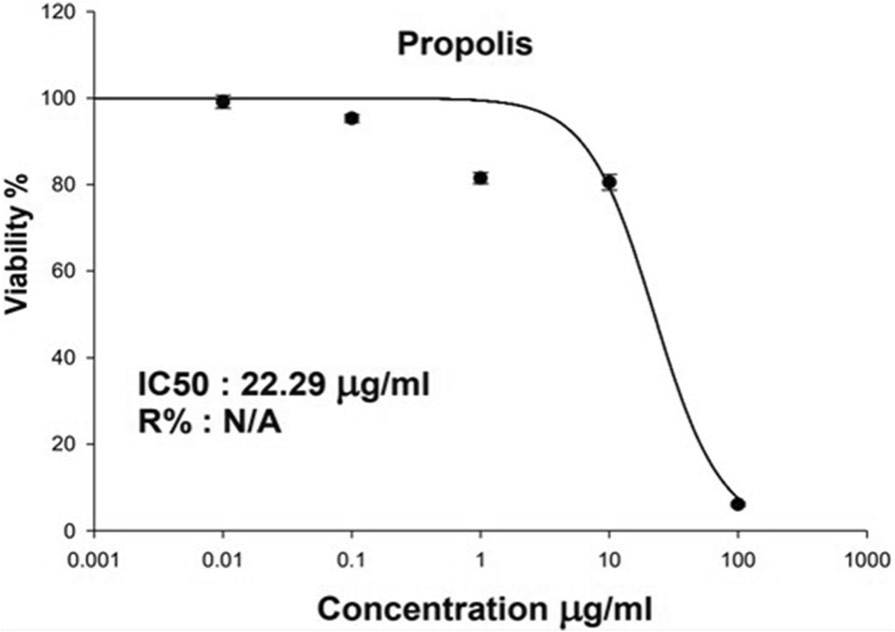
Determination of the IC_50_ of propolis nano-emulsion following 72 h incubation of Human Skin Fibroblast (HSF) in various concentrations and analyzed using SRB assay

**Fig. 8:**
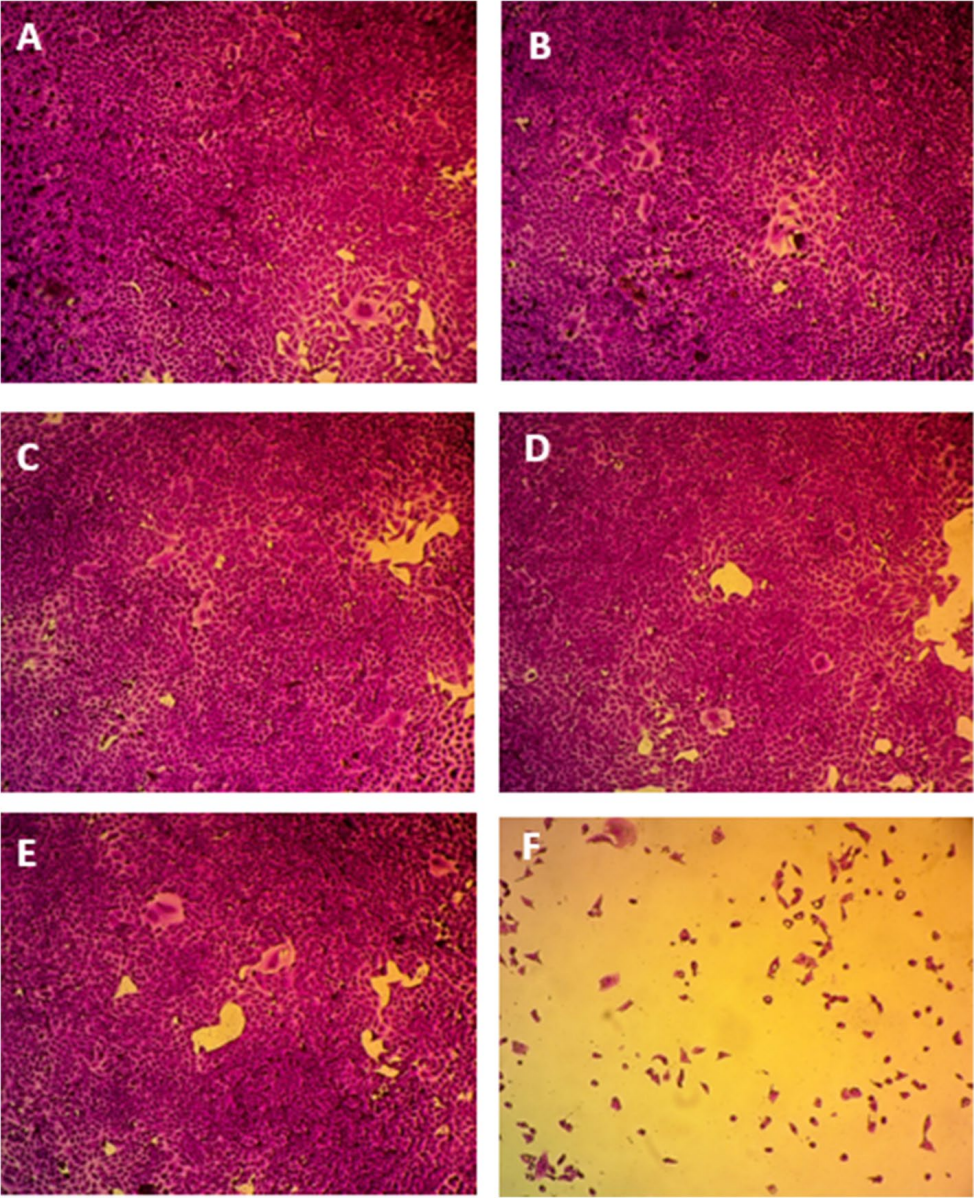
Microscopic images reveal the effect of various concentrations of propolis nano-emulsion on cell viability using SRB assay. **A** Control, **B** 0.01 μg/ml, **C** 0.1 μg/ml, **D** 1 μg/ml, **E** 10 μg/ml, **F** 100 μg/ml

Based on the assessment of cellular protein content, the Sulforhodamine B (SRB) test has been designated to be used in a 96-well configuration for drug toxicity testing on adherent cells. The test depends on SRB’s capacity to bind proteins of cells that have been trichloroacetic acid-fixed on tissue-culture plates [56]. The prepared NE showed an IC_50_ of 22.29 μg/ml against Human Skin Fibroblast cell line **(**Fig. 7**)**. The change in cell viability was as the follows: (99%), (95%), (81%), (80%), and (6%) at the concentrations of (0.01 μg/ml), (0.1 μg/ml), (1 μg/ml), (10 μg/ml), and (100 μg/ml), respectively **(**Fig. 8**)**. The cytotoxicity of the propolis emulsion can be observed at the cellular level, where the increase in concentration was inversely proportional to the cell viability**.** Such cytotoxicity effect could be attributed to the presence of Tween 80 that was used as surfactant [57] in the NE fabrication or due to presence of hyaluronic acid [58]; as a previous study conducted by Shehata et al. [59] who indicated the safety of propolis from various geographical origins (including Egyptian propolis) on human dermal fibroblasts; IC_50_ values of all propolis samples were above 300 μg/ml. Thus, it is highly unlikely for the propolis to be the cytotoxic constituent in the NE.

### DPPH assay

**Fig. 9:**
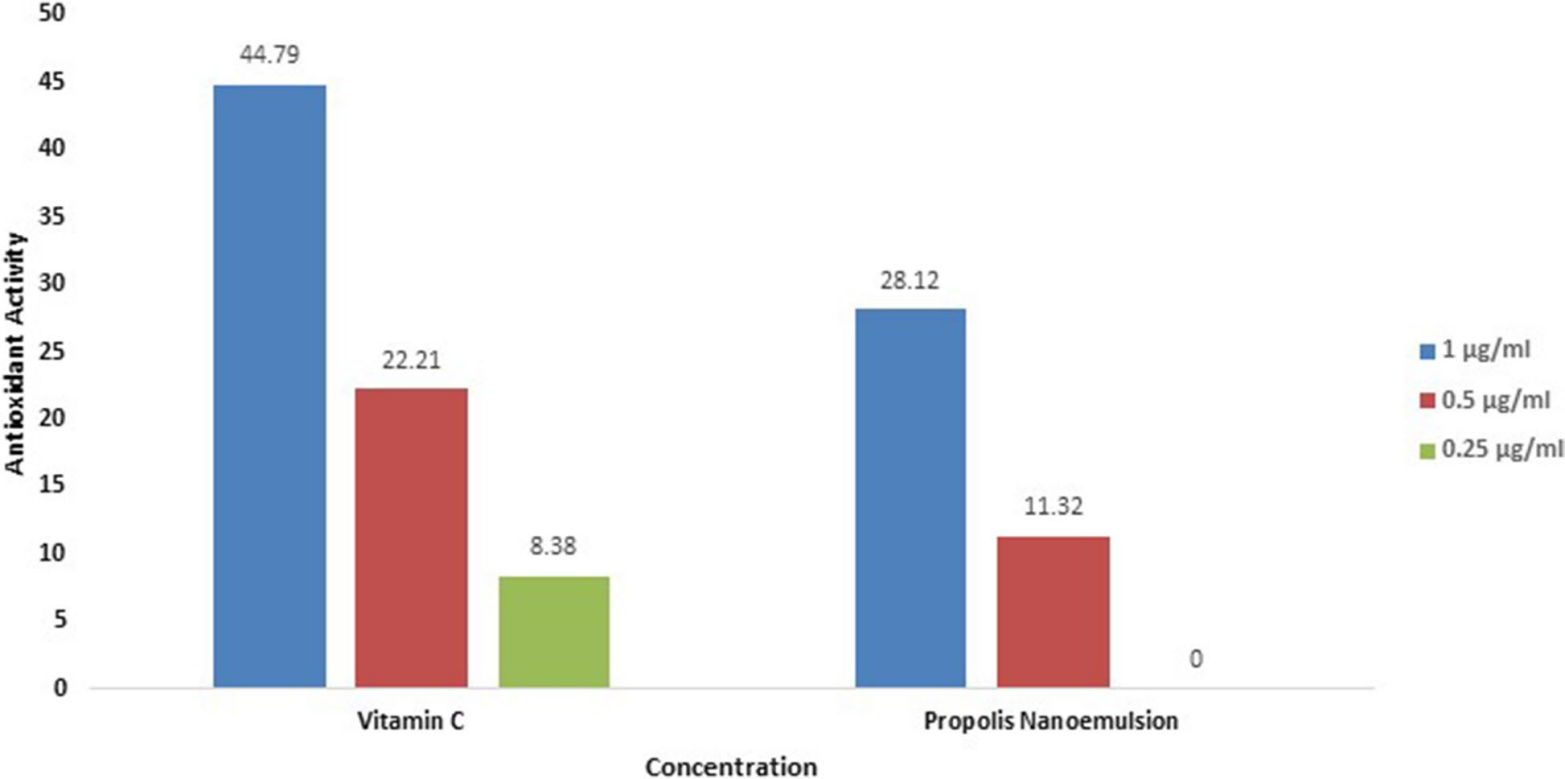
Antioxidant activity of propolis nano-emulsion

A recent study on the role of antioxidants in wound healing by [60] found that antioxidant compounds that regulate non-toxic ROS levels in wound tissues could accelerate the healing. This was also supported by another study conducted by Sahib et al. [61] on the impact of antioxidants on burn patients’ risk of wound infection, which revealed a lower incidence of wound infection, faster healing time, and improved mortality rate in burn patients treated with antioxidants compared to patients who did not receive antioxidants as part of their treatment plan. DPPH test is one of the most widely used colorimetric assays to determine the radical-scavenging ability of plants and extracts. This technique is based on the synthetic and reliable radical DPPH. DPPH loses its free radical characteristic and turns yellow when it interacts with an antioxidant molecule [62]. In the present study, the propolis NE was found to have an antioxidant activity of 28.12% at concentration of 1 μg/ml and 11.32% at concentration of 0.5 μg/ml. On the other hand, the control (Vitamin C) showed antioxidant activities of 44.79, 22.21, and 8.38% at concentrations of 1, 0.5 and 0.25 μg/ml, respectively (Fig. 9**).** The achieved antioxidant potential may be due to the presence of propolis as main component in the NE.

### Assessment of the antimicrobial activity

The antimicrobial activity of the prepared NE against *Staphylococcus aureus* (ATCC 25923) revealed that the Minimum Inhibitory Concentration MIC was 2.5 mg/ml and the Minimum Bactericidal Concentration MBC was 5 mg/ml**.** As for *Escherichia coli* (ATCC 8739), the NE had a MIC of 5 mg/ml and MBC of 10 mg/ml. In addition, it displayed an antimicrobial effect on *Pseudomonas aeruginosa (*ATCC 27853), which had MIC and MBC of 2.5 mg/ml and 5 mg/ml, respectively.

Dilution methods are the standard techniques for antimicrobial susceptibility testing which are used to calculate the minimum inhibitory concentrations and minimum bactericidal concentration of potential antimicrobial drugs [63]. In the current study, the propolis NE showed a strong antimicrobial activity against *Staphylococcus aureus* (ATCC 25923), *Escherichia coli* (ATCC 8739), and *Pseudomonas aeruginosa* (ATCC 27853). This agreed with a study conducted by Archin et al. [64] in which they prepared NE from an ethanolic extract of propolis. The study revealed that the propolis NE had an antimicrobial effect against *Pseudomonas aeruginosa*. Our results also agreed with another study conducted by Seibert et al. [33] as they developed a propolis NE for the utilization of its potential antimicrobial activity as a natural preservative. They tested the NE against multiple gram-negative and gram-positive bacteria and revealed that *Staphylococcus aureus* was the most susceptible to the NE. Furthermore, in a study performed by Al-Ani et al. [65] on the antimicrobial activity of European propolis from various geographical locations on 42 strains of microorganisms, they found that propolis had a strong antimicrobial activity against *Escherichia coli*.

### Wound scratch assay

**Fig. 10:**
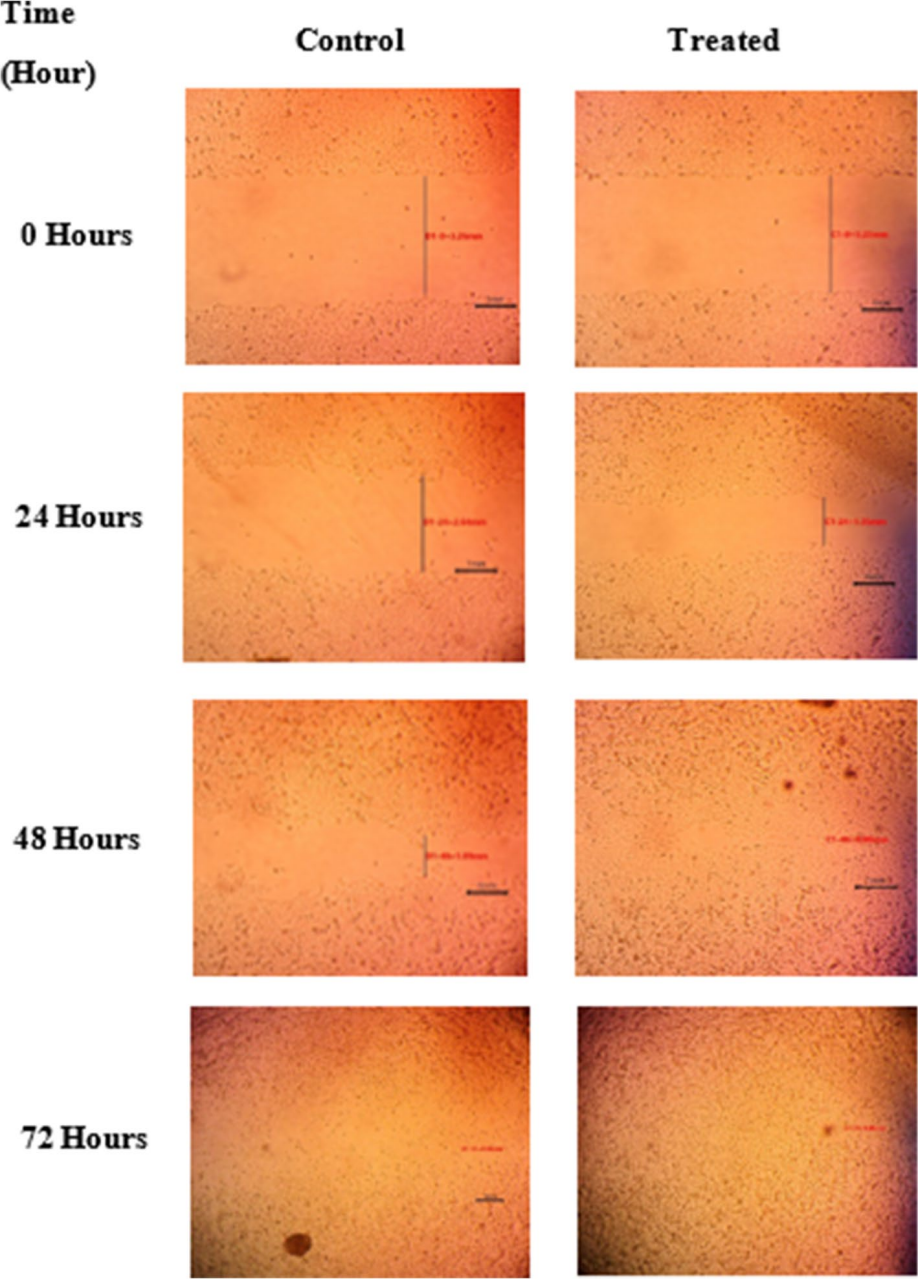
Effect of propolis nano-emulsion on cell migration in human skin fibroblasts

**Fig. 11:**
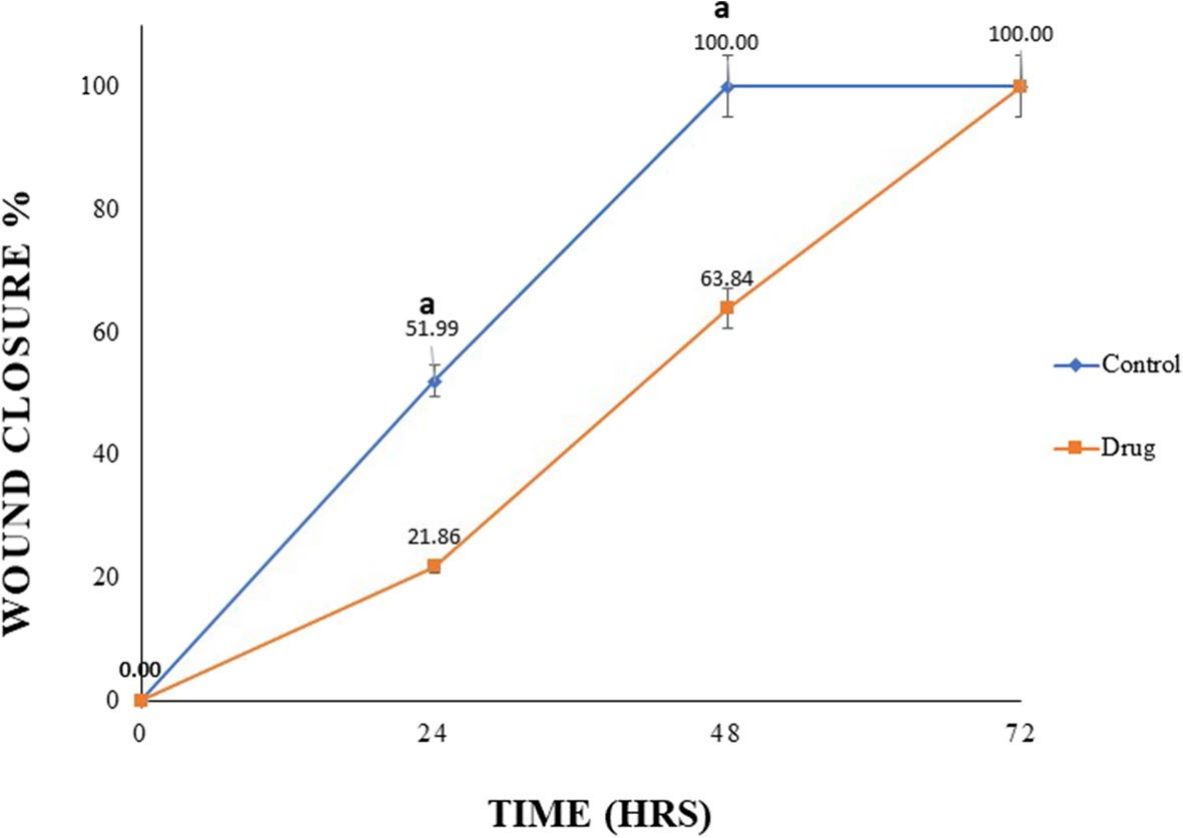
*In-vitro* wound healing activity of propolis nano-emulsion presented as a percentage of wound closure at various time intervals. (a) Mean significant difference when compared with the control when *p* ≤ 0.05

The formulated propolis NE was assessed for its *in-vitro* wound healing properties using cell migration assay on HSF cell line. By using the microscopic images in Fig. 10, it was possible to evaluate the evolution of the gap created in the confluent cell monolayer in the presence of the NE. There was a significant increase (*P* < 0.05) in the wound closure percentage at both 24 and 48 h in the HSF cells that were treated with the 10 μg/mL propolis NE when compared with the control (Fig. 11). However, there was no significant difference between the treated cells and the control cells at 72 h.

Dermal fibroblasts are the primary cell type in the connective tissue of skin; they are crucial in the bioengineering of skin and the healing of wounds [66]. Due to their crucial involvement in the creation of granulation tissue, fibroblast migration and proliferation are necessary and rate-limiting stages for repairing wounds [67]. Therefore, the present study evaluated the wound healing capabilities of the formulated propolis NE using cell migration assay on normal human dermal fibroblast cell line. Cell migration assay, also known as scratch assay, is a common in vitro method for examining collective cell migration in two dimensions. In this test, a confluent monolayer is physically excluded from the surrounding region, or the cells are driven out of the area by mechanical, thermal, or chemical damage. The cells’ migration is then observed, and the rate of gap closure is calculated [68].

In the current study, the propolis NE showed a significant (*P* ≤ 0.05) increase in the wound closure percentage on human skin fibroblasts compared with the control. The propolis NE achieved complete gap closure within the first 48 h, while the control cells achieved gap closure after 72 h. Our results agreed with a study conducted by Ebadi and Fazeli [69] who evaluated the *in-vitro* wound healing effect of propolis on human dermal fibroblasts as they revealed that at the tested concentrations, the propolis significantly increase the cell migration during the first and second 24 h. In another study performed by Martinotti and Ranzato [70] on the possible impact of propolis on wound healing through the modulation of Aquaporin (AQP3) expression levels, their scratch assay findings demonstrated that propolis boosted keratinocytes’ ability to repair the wounds. They noticed that propolis had stronger chemotactic effects than 20% platelet lysate.

### *In-vivo* evaluating wound healing properties

**Fig. 12:**
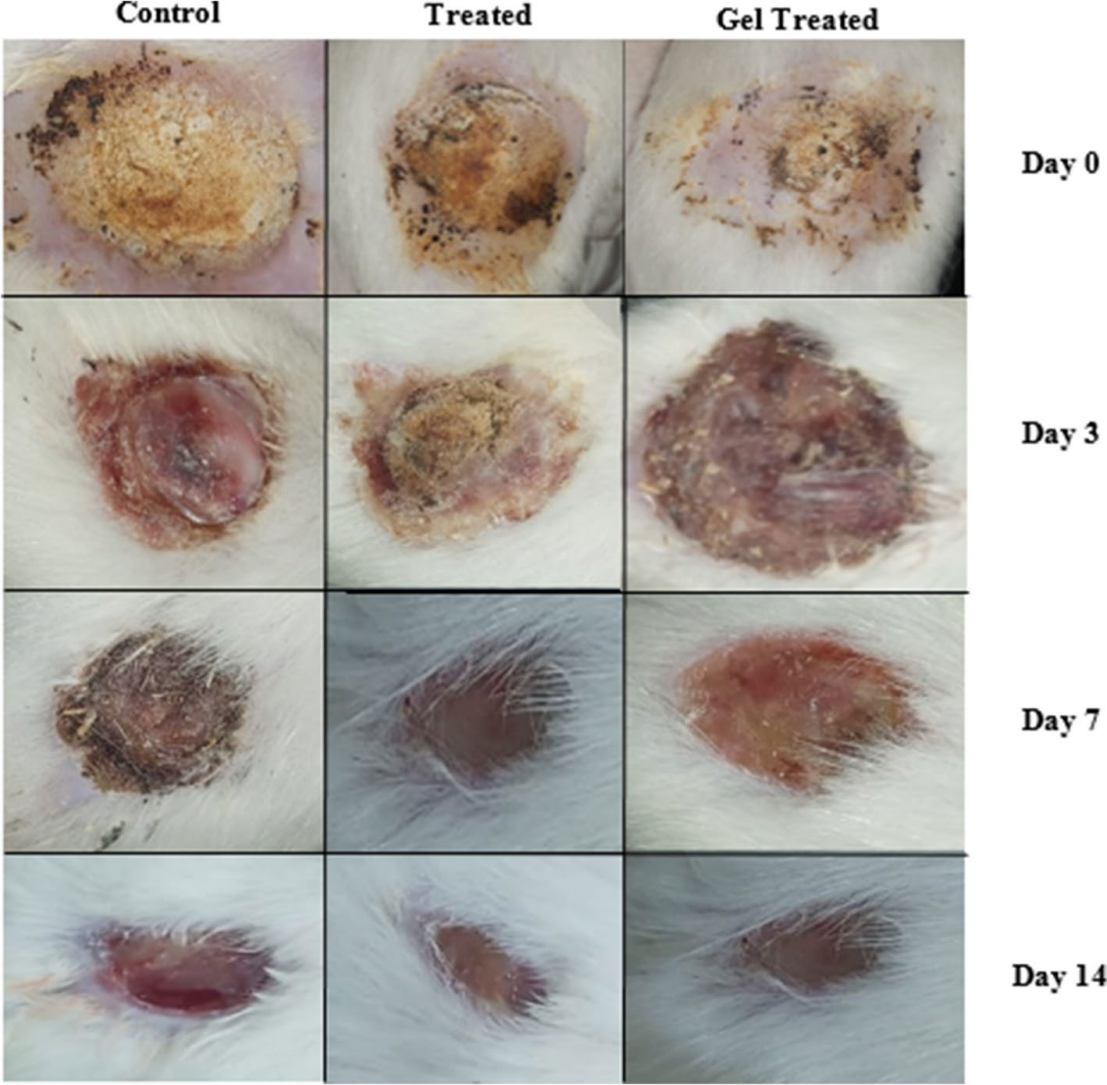
Burn areas in rats were topically treated with the propolis nano-emulsion compared with the group treated with the gel form of the nano-emulsion and untreated rats (Control) at days 3, 7 and 14

**Fig. 13:**
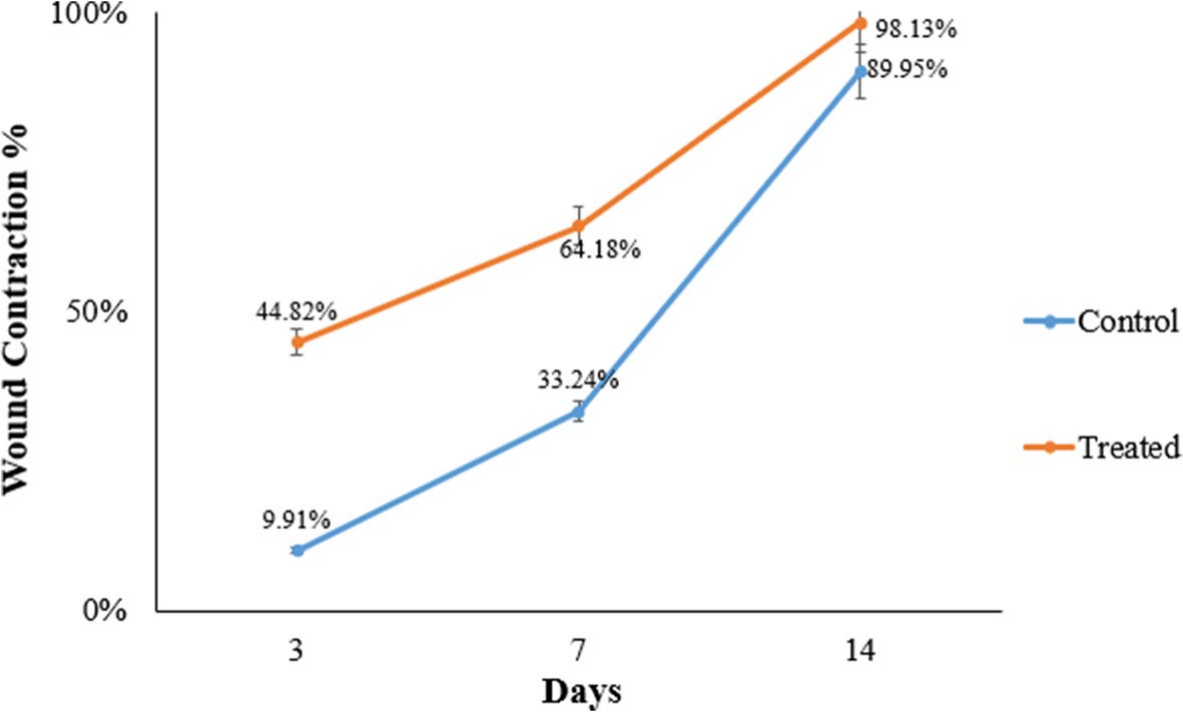
*In-vivo* wound contraction percentage induced by the formulated propolis nano-emulsion and gel at different time intervals compared to the control. (a) Mean significant difference when compared with control group when *p* ≤ 0.05

The treated group displayed a significant decrease in wound surface area on the 3rd, 7th, and 14th day post-treatment **(**Fig. 12). This was apparent in the exponential increment in wound closure percentage (WC%) of the treated group during the first week of treatment, as opposed to the control group **(**Fig. 13). The results of the present study agreed with a study conducted by Pessolato et al. [71]**,** as they evaluated the role of propolis ointment on second-degree burns in rats. They reported that the propolis therapy expedited tissue regeneration and reduced local inflammation. Furthermore, propolis treatment promoted collagen fiber formation during the period between the 14th and 21st days. Additionally, they highlighted how propolis might help with wound debridement.

Also on the 14th day post-treatment, there was less scar formation was detected with complete closure of wounds which appeared as normal skin. At the same time interval, the control group showed scabs and a dark red appearance of the burns.

The promotion of wound closure can also be attributed to not only the propolis, but also the hyaluronic acid in the NE. This was supported by a recent study carried out by Ciccone et al. [72] who compared the wound healing effects of two hyaluronic acid preparations on both fibroblasts and endothelial cells, as the result of scratch assay revealed that hyaluronic acid significantly promoted fibroblast migration with reduction of the wound area.

The hypothesis is that the observed changes in extracellular matrix composition following the topical application of propolis may be owed to its capacity to stimulate wound bed matrix remodeling through the minimization of lipid peroxidation and repression of cell necrosis. As well as the increased extractability of collagen type III [73]. Additionally, by encouraging the synthesis of the glycosaminoglycans (chondroitin/dermatan sulfate and hyaluronic acid) in the wound bed, which are essential for granulation, tissue development, and wound closure, it speeds up the healing of burnt tissue [74].

Besides the role of propolis in wound repair, hyaluronic acid in NE is also an important agent in the process of healing. When hyaluronic acid is applied to the wounds, water retention is improved, promoting favorable conditions for the production of collagen and elastin and enabling cell proliferation and differentiation, resulting in a quicker time for wound healing and histological attributes like improved elasticity and higher microvascular density. Hyaluronic acid’s anti-inflammatory capabilities also have an impact on wound healing by inhibiting the conversion of the wound and the development of keloids or hypertrophic scars [75].

Furthermore, the vitamin K which present in the formulated nano-emulsion was contributed to increasing the overall efficiency of wound repair. This was supported by a randomized controlled trial conducted by Pazyar et al. [31] on the wound healing effects of topical vitamin K. Their results showed a significant increase in wound contraction and faster healing time for patients treated with topical vitamin K. In another study performed by Osman and Amin [30] evaluating the wound healing effect of vitamin K in comparison with the commonly used wound dressing agents, they found that the best wound contraction percentage (99%) was achieved by the vitamin K-treated rat group, as a result of the significant increase in TGF-β and PDGF levels.

### Histopathological analysis

**Fig. 14:**
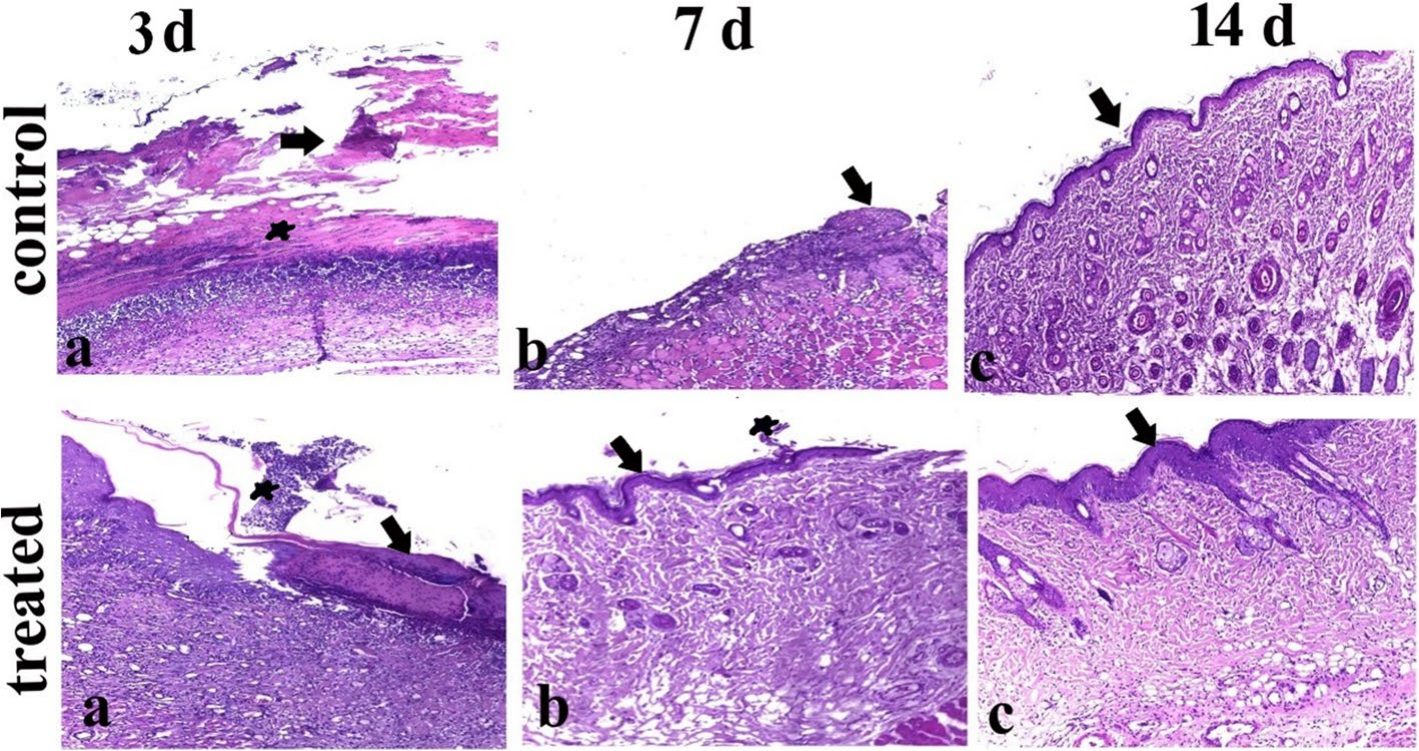
Light photomicrograph of the skin burn of the control and treated group at the 3rd, 7th, and 14th days sectioned with H&E.: where control group showing a wide gap with complete destruction of superficial skin layers (arrow), besides edematous fluid (star) at 3rd (**a**), while necrotic mass at the attached to wound at 7th day (arrow) (**b**); whereas at 14th day, reepithelization with granulation tissues formation occurred (arrow) (**c**). Meantime treated group showing collection edematous mass adherent to wound tissues (arrow), also necrosed and sloughed tissues with inflammatory infiltration (star) at 3rd day (**a**), whilst at 7th day; partial reepithelization occurred (arrow) with few necrotic debris (star) (**b**), consequently complete reepithelization with full granulation tissues formation covered with epithelium at 14th day (arrow) (**c**)

As shown in Fig. 14, the Histopathological examination revealed a wide gap with complete destruction of superficial skin layers, besides edematous fluid, in the control group on the third day, while necrotic mass attached to the wound was examined on the 7th day. Whereas on the 14th day, re-epithelization with granulation tissues formation started to appear. Meanwhile, the treated group demonstrated a collected edematous mass adhered to the wound tissue, as well as inflammatory cells infiltration on the 3rd day, while partial re-epithelization with little necrotic debris occurred on the 7th day, consequently complete re-epithelization with full granulation tissues formation covered with epithelium was appeared on the 14th day post-treatment.

**Fig. 15:**
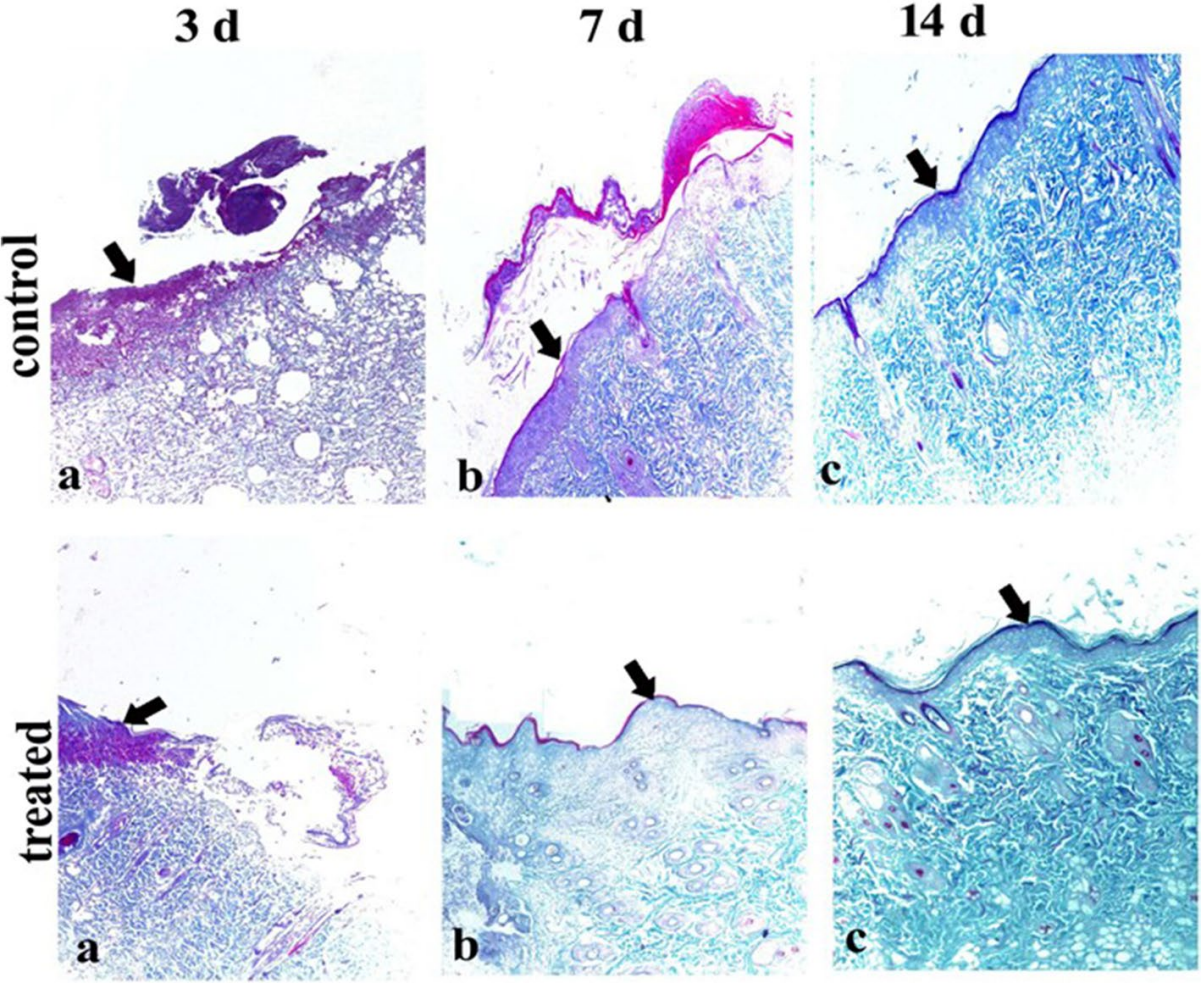
Light photomicrograph of the skin burn of the control and treated group at the 3rd, 7th, and 14th days sectioned with Crossman trichrome:: no granulation tissue formed at 3rd days pretreatment (arrow), while mild granulation tissue begin to appear at 3th days post-treatment (arrow), granulation tissue start at the base of one edge at 7th days pretreatment (arrow), granulation tissue fill all the wound gap and center with epithelization at 7th days post-treatment (arrow), while mature fibrous C.T. with shrinkage of granulation tissue at 14th days pre and post treatment (arrows). Notes: Collagen is stained blue-green, while cytoplasm, red blood cells, and nuclei are stained red

The burns of control and treated groups on the 3rd, 7th, and 14th days stained with Crossman trichrome (Fig. 15). showed no granulation tissue formation on the 3rd day post-treatment, while mild granulation tissue began to appear on the 7th day post-treatment which started at the base of one edge and fill all the wound gap and center with epithelium, while mature fibrous C.T. with shrinkage of granulation tissue appeared on the 14th days post-treatment.

The histological scores of wound healings; fibrous tissue formation, cell mitosis, tissue granulation, re-epithelization, Angiogenesis, fibroblast, and neovascularization were increased in the treated group compared with the control. At the same time, ulcerative necrosis with cell debris, inflammatory edema, and inflammatory cell infiltration were increased in the control group than the treated group (Table 1).

**Table 1:** Histological score of skin burn of control and treated group at different stages stained with H&E., and Crossman’s trichrome graded according to severity into severe (+++), moderate (++), mild (+) and absent (−)

Groups						
Lesions	Control			Treated		
	3rd d	7th d	14th d	3rd d	7th d	14th d
**Haematoxylin and eosin**						
**Fibrous tissues formation**	–	+	++	–	+	+++
**Cell mitosis**	–	+	++	+	++	+++
**Tissues granulation**	–	+	++	+	++	++
**Re-epithelization**	–	+	++	+	+	++
**Angiogenesis**	–	+	++	–	+	+++
**Fibroblast**	–	+	+	+	+	++
**Neovascularization**	–	+	++	+	++	++
**Ulcerative necrosis with cell debris**	+++	++	+	++	+	–
**Inflammatory edema**	+++	+++	+	+++	+	–
**Inflammatory cells infiltration**	++	++	+	++	+	–
**Crossman’s trichrome**						
**Extracellular matrix**	–	+	++	–	+	+++
**Fibroblast**	–	+	++	–	++	+++
**Collagen fibers**	–	+	++	+	++	+++
**Blue-green stained coloration of dense collagen fibers**	–	+	++	–	++	+++

Severe (+++), moderate (++), mild (+) and absent (−)

## Conclusions

It could be concluded that the formulated propolis NE had a mesh-like structure with a size range of 80 to 180 nm and a negative surface charge of (− 21.6 ± 6.22 mV), indicating colloidal stability. The NE had an IC_50_ of 22.29 μg/ml on human skin fibroblast cell line. It also had an antioxidant activity of 28.12% at concentration of 1 μg/ml. Moreover, the NE displayed powerful antimicrobial activity against (*Escherichia coli, Pseudomonas aeruginosa* and *Staphylococcus aureus*), commonly isolated from burns. Moreover, the propolis NE significantly promoted cell migration/wound closure *in-vitro* on human skin fibroblast cell line. Furthermore, a significant increase in the wound contraction percentage was observed in the 2nd degree burns of rats treated with the propolis NE compared with the control during the 14 days of the *in-vivo* experiment. Thus, the formulated propolis NE showed an effective antimicrobial and healing activities in the treatment of second-degree burns.

### Limitation of the study

Future investigations for the identification of the cellular mechanisms of wound healing induced by the NE are recommended. This will allow for a better understanding of the mode of action of NE in ameliorating the cellular damage caused by burns and how it enhances the wound healing process. Thus, paving the way for human trials someday.

## Data Availability

Data for this study is available inside the manuscript.
